# Supplementary figures and images for: Detection of Mycobacterium tuberculosis from tongue swabs using sonication and sequence-specific hybridization capture
Source: PLoS One. 2024 Aug 15;19(8):e0308235. doi: 10.1371/journal.pone.0308235 (PMC11326604; doi:10.1371/journal.pone.0308235)

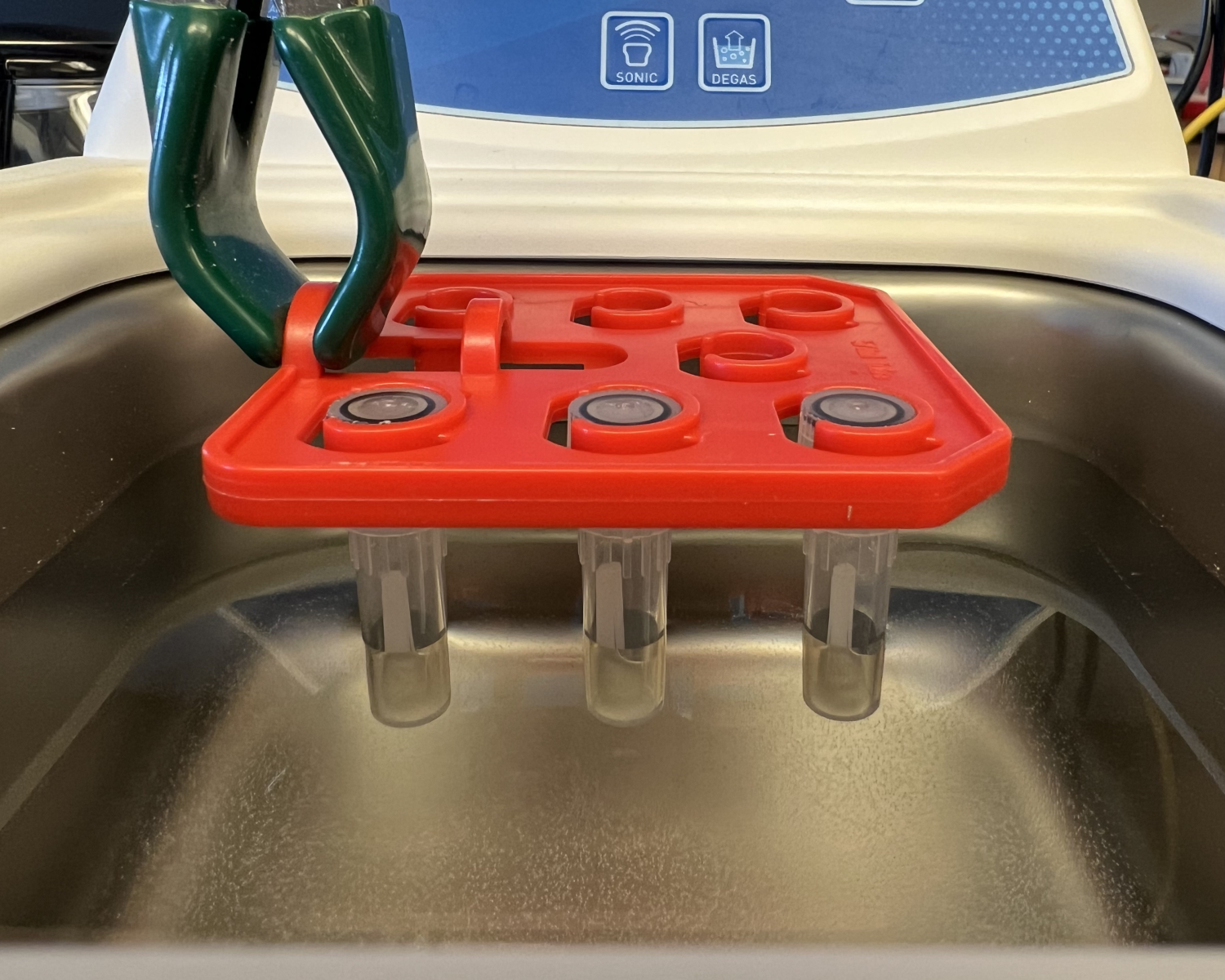

Supplement: S1 Fig — (TIF) [file pone.0308235.s001.tif]

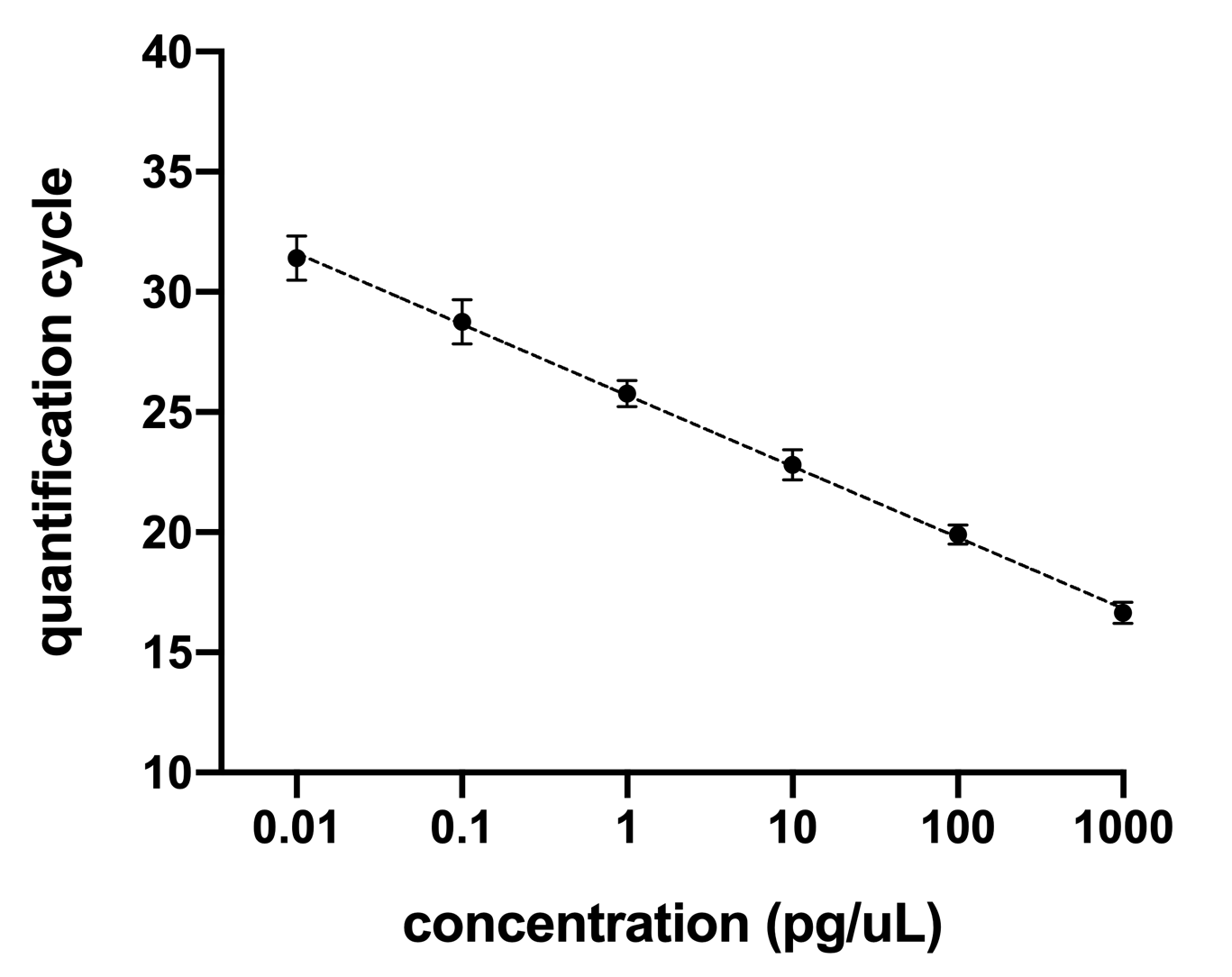

Supplement: S2 Fig — Representative calibration curve illustrating qPCR standards across a range of concentrations from 0.01–1000 pg/μL (0.05–5000 pg/rxn) of purified MTB DNA (mean ± SD, n = 7). (TIF) [file pone.0308235.s002.tif]

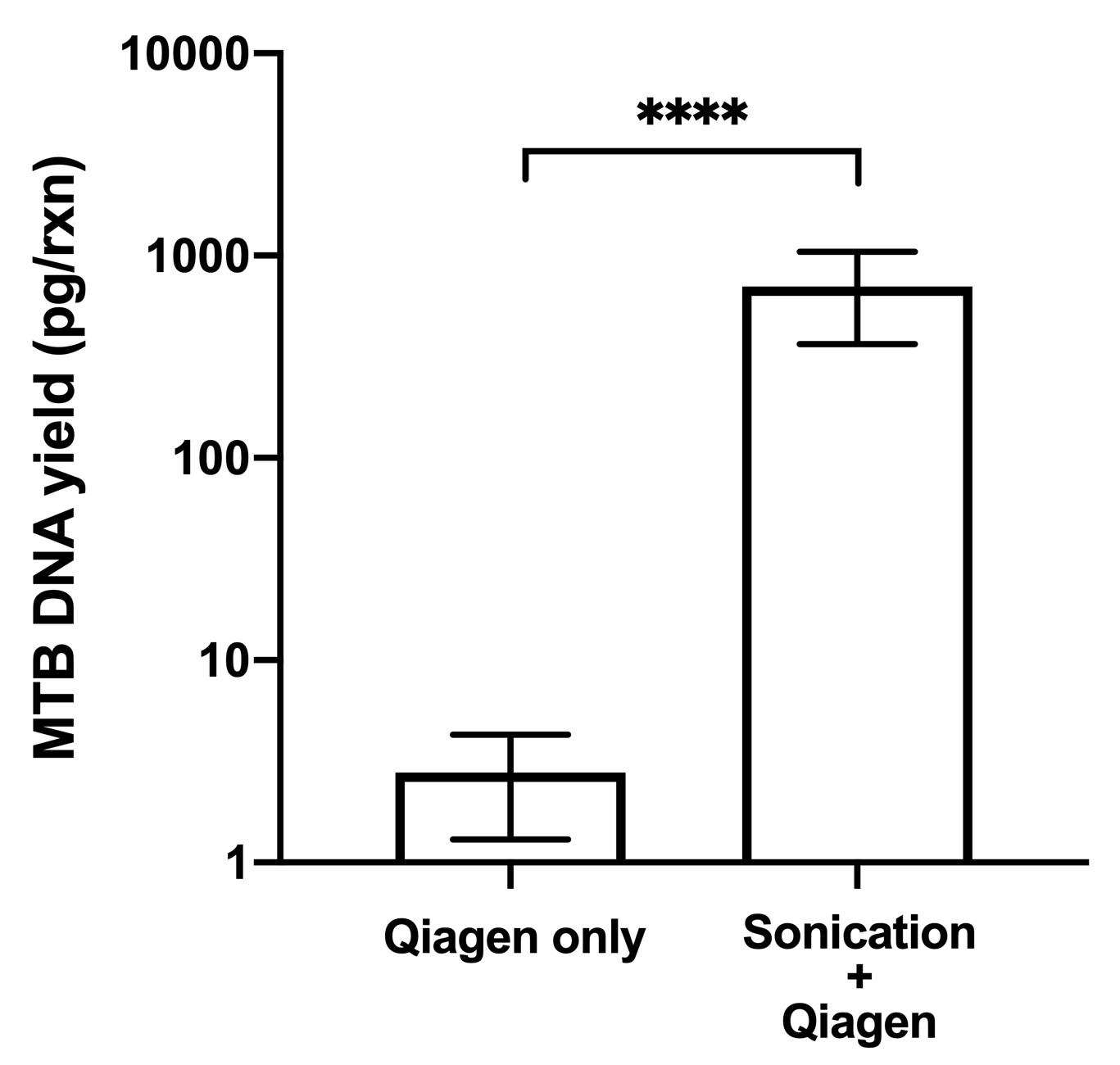

Supplement: S3 Fig — The QIAamp DNA extraction kit was performed on tongue swabs spiked with 104 H37Ra MTB cells both with and without an upstream sonication lysis step. MTB DNA yield was significantly higher when sonication was included. **** indicates P value of < 0.0001 (two-sample t-test). Data is presented on a logarithmic scale (mean ± SD, n = 3). (TIF) [file pone.0308235.s003.tif]

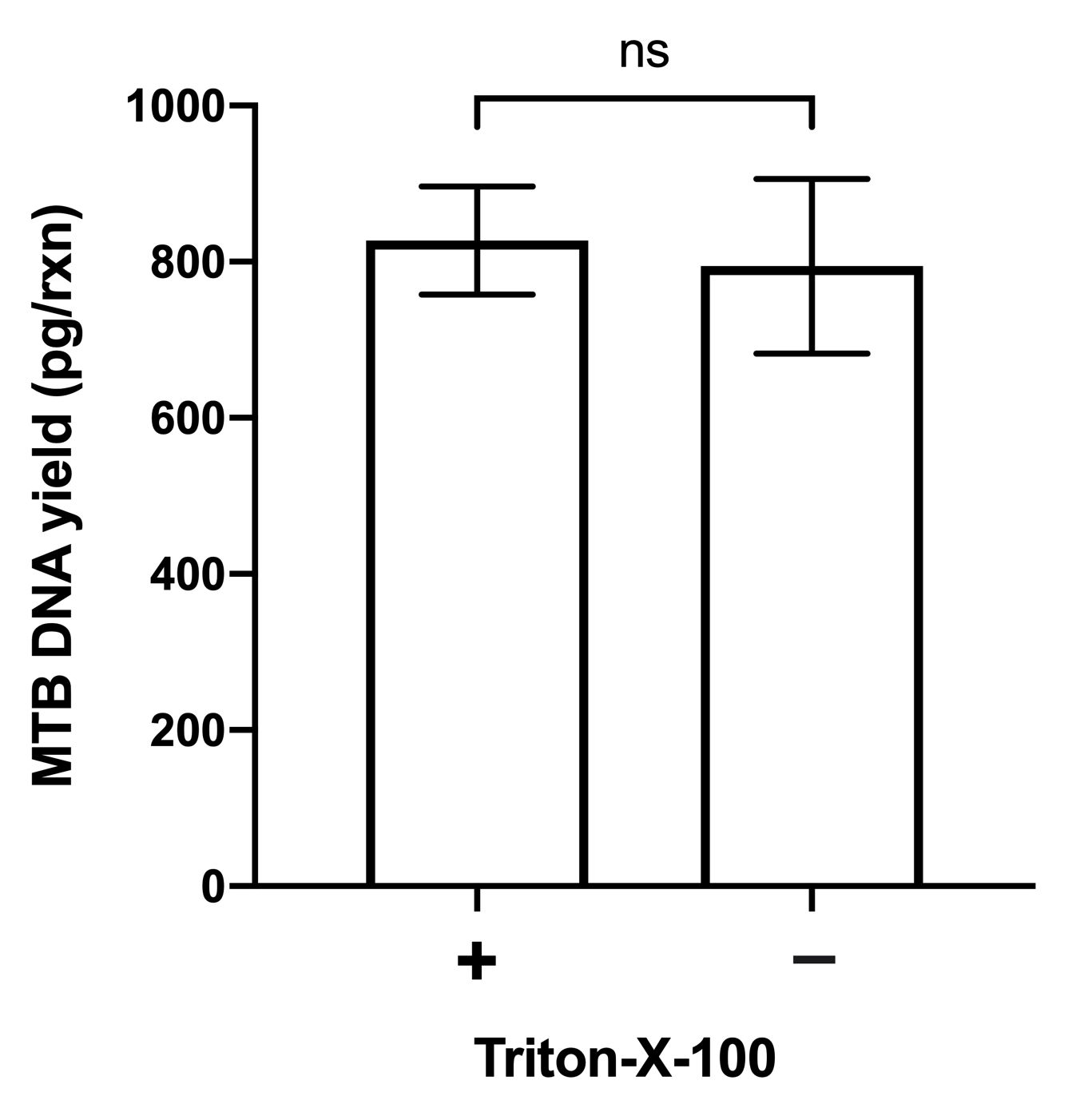

Supplement: S4 Fig — Hybridization capture was performed on tongue swabs spiked with 103 H37Ra MTB cells and resuspended in either TE buffer or lysis buffer (TE, 1% Triton-X-100). There was no significant difference in MTB DNA yield between the two groups (mean ± SD, n = 3). NS indicates P-value > 0.05 (two-sample t-test). (TIF) [file pone.0308235.s004.tif]
