# Supplementary material for: Detection of Mycobacterium tuberculosis from tongue swabs using sonication and sequence-specific hybridization capture
Source: PLoS One. 2024 Aug 15;19(8):e0308235. doi: 10.1371/journal.pone.0308235 (PMC11326604; doi:10.1371/journal.pone.0308235)
Supplement: Attachment — Submitted filename: Response to Reviewers.docx [file pone.0308235.s006.docx]

**Response to Reviewers**

Dear PLOS ONE reviewers,

Thank you for the opportunity to resubmit our manuscript titled “Detection of *Mycobacterium tuberculosis* from tongue swabs using sonication and sequence-specific hybridization capture” for publication in PLOS ONE. We appreciate the time and effort dedicated to providing feedback on our manuscript, and we have made the follow changes to our submission to address the feedback provided.

**Reviewers' Comments to the Authors:**

“Specifically, the ethics approval documents you provided do not cover the full research period mentioned in the Methods section (8 June 2021 to 30 April 2022). It is required ethics approval documentation should demonstrate that ethics approval was obtained to cover the entire duration of the study. You only provided documents to support the approval from 17 March 2020 to 30 March 2021; and 2/22/2023 to 2/21/2024.”

**Author response:** Thank you for pointing out that the previously submitted ethics approval documents did not fully cover the full research period. We have addressed this issue by attaching three documents which support ethics approval for the following time periods: 4/10/2020 to 4/9/2021, 3/26/2021 to 3/25/2022, and 3/17/2022 to 3/16/2023. We believe that these documents satisfactorily demonstrate ethics approval during the entire duration of the study.
