# Supplementary material for: Detection of Mycobacterium tuberculosis from tongue swabs using sonication and sequence-specific hybridization capture
Source: PLoS One. 2024 Aug 15;19(8):e0308235. doi: 10.1371/journal.pone.0308235 (PMC11326604; doi:10.1371/journal.pone.0308235)
Supplement: Attachment — Submitted filename: Response_To_Reviewers_2024_01_28.docx [file pone.0308235.s007.docx]

**Response to Reviewers**

Dear PLOS ONE reviewers,

Thank you for the opportunity to resubmit our manuscript titled “Detection of *Mycobacterium tuberculosis* from tongue swabs using sonication and sequence-specific hybridization capture” for publication in PLOS ONE. We appreciate the time and effort dedicated to providing feedback on our manuscript, and we have made the following changes to our submission to address the feedback provided. These changes are found in the revised manuscript file with tracked changes. Please see below, in blue, for a point-by-point response to the reviewers’ comments and concerns. All line numbers refer to the clean revised manuscript file **without** tracked changes.

**Reviewers' Comments to the Authors:**

**Reviewer # 1:**

**Reviewer comment 1**: *In ‘Materials and Methods’ section, under the heading ‘Methods for DNA extraction in spiked oral swabs,’ subheading ‘Quantification by qPCR,’ you mentioned a standard curve of purified MTB DNA was used for conversion of Cq values to DNA quantity, but the same was not included in the document. It could provide a reference for the range of DNA quantity versus Cq values you have used in the study.*

**Author response**: Throughout our study, multiple qPCR reactions were performed, each accompanied by its own standard curve. Due to space limitations and to avoid redundancy, we have chosen to include one representative standard curve (S2 Fig) as a reference for the reader. We believe this new supplemental figure adequately captures the range of the DNA quantity used to create the standard curves in this study. We appreciate this helpful suggestion.

**Reviewer comment 2**: *In ‘Fig 1. Workflow of MTB DNA extraction from oral swabs,’ it would be beneficial if you could include in the figure the temperature and duration you have used for individual steps*

**Author response**: We appreciate your suggestion regarding the addition of temperature and duration information for individual steps in the figure. However, we intentionally omitted this information from the figure to maintain clarity and prevent visual clutter. Our objective with this figure was to provide a concise overview of the main steps in the hybridization capture protocol. For readers who want to see the detailed temperature and duration for each step, we have included this information in the figure description of Fig 1 (lines 118–124).

**Reviewer comment 3**: *In ‘Materials and Methods’ section, under the heading ‘Clinical sample evaluation methods’, subheading ‘collection of clinical samples and test assignment,’ you mentioned that participants were in two cohorts, however, you did not mention the number of participants in each cohort included and the impact of the two cohorts will have on the conducted study.*

**Author response**: In response to the suggestion from one of the reviewers, we have put together a STARD diagram (Fig 2) that should clarify to the readers the number of participants in each cohort and how samples from each cohort were used. Changes to the text have been made to clarify which cohorts were involved in the sensitivity and specificity calculations, including the addition of lines 220–221, which now states: “All negative by Xpert Ultra samples included in this study are therefore from Cohort 2.”

Regarding the impact on the study, the study was not constructed to compare Cohort 2’s participants, who were initially presumptive TB cases but later confirmed negative for TB, to Cohort 1’s participants, who were all confirmed-TB cases. Both cohorts share the same enrollment sites, time periods partially overlapped, and participants were all voluntarily healthcare seeking. Due to Cohort 1’s structure of collection and enrollment (designed for a primary study different to this one) we would not have had any clinically negative confirmed samples to test. The inclusion of negative samples from Cohort 2 enhances the robustness of our study. No samples confirmed positive for TB from Cohort 2 were included in the analyses.

**Reviewer comment 4**: *In ‘Materials and Methods’ section, for preparation of spiked oral swabs for hybridization capture, swab head was heated to 100 °C for 10 mins in lysis buffer, but for preparation of spiked oral swabs for QIA-EtOH and clinical oral swabs for hybridization capture and QIA-EtOH you heated the swab to 100 °C for 10 min and then TE buffer or lysis buffer was added. Won’t heating in the buffer and heating without the buffer impact the quantity of DNA extracted and the accuracy of the extraction process?*

**Author response**: The hybridization capture protocol was optimized for heating the swab in lysis buffer because we initially hypothesized that this would improve DNA yield. However, subsequent experimentation revealed that there was no significant difference in DNA yield between heating in buffer and heating without buffer when applied to spiked samples (data not shown here). We speculate that any potential drawbacks associated with heating the swabs without buffer were mitigated by the robust lysis performance of sonication.

In the case of experiments conducted on spiked samples using QIA-EtOH, we followed the established protocol used by our consortium (e.g. Luabeya et al, 2019 [1]). However, the clinical swabs we received were stored dry and required an inactivation step (heating to 100 °C) before opening for biosafety reasons. Therefore, we performed the heating step without buffer in these samples. Although the modification to heat the swabs without buffer in the clinical samples may have affected DNA extraction efficiency, biosafety concerns overrode this issue.

**Reviewer comment 5**: *In ‘Results’ section, under the heading ‘Comparison of hybridization capture to Qiagen DNA extraction and ethanol precipitation in MTB-spiked oral swabs,’ the average DNA yield for spiked oral swabs by hybridization capture was mentioned as 2200 ± 1600 pg/rxn. A variation of ± 1600 pg is very large, you did not mention the reason for such variation.*

**Author response**: While we respectfully acknowledge the concern about this high standard deviation, we believe it is important to clarify that this variation is within the expected range based on our experimental conditions.

Various sources of error may contribute to this observed variation, as discussed in the manuscript. Notably, donor swab variability plays a significant role. As mentioned in lines 413–417, high concentration of biotin in certain swabs may compete with biotinylated capture probes for binding to magnetic beads, leading to decrease in DNA yield. Furthermore, other factors including presence of food particles, nucleases, or other inhibitors in swab samples could lead to variable DNA recovery.

**Reviewer comment 6**: *Also, for sample matrix effect and sonication effect measurement you used Oral swabs spiked with 103 MTB cells but for Comparison of hybridization capture to QIA-EtOH DNA extraction you used oral swabs spiked with 104 MTB cells. The document does not include the reason for this increase in the number of MTB cells for spiking.*

**Author response**: In our experiments investigating the effects of sample matrix and sonication, we intentionally spiked samples with 10^3^ MTB cells. This lower cell count was selected to better replicate the lower quantity of MTB cells expected to be found on many clinical swabs.

In contrast, when comparing hybridization capture to QIA-EtOH, we increased the spiked cell count to 10^4^ MTB cells. This decision was influenced by our anticipation, derived from prior experiments, that QIA-EtOH would perform sub-optimally in spiked swabs. The higher cell count in this comparison was chosen to minimize the risk of qPCR drop-offs for these specific samples. We believe that these variations in spiking quantity were crucial to addressing the specific objectives of each experimental set. To provide clarity on this for the reader, we have included this reasoning in the manuscript (lines 323–325) which states: “Swabs were spiked with 10^4^ MTB cells (as opposed to 10^3^ previously) because we anticipated poor performance from QIA-EtOH, and we aimed to quantify samples by qPCR in this group.”

**Reviewer comment 7**: *In the ‘Fig 4. Hybridization capture improves DNA yield over QIA-EtOH in MTB-spiked samples,’ the bar for sonication alone was drawn bigger than the value it was representing 27 ± 24 pg/rxn and the DNA yield value for 100°C boil is not included in the figure description.*

**Author response**: Thank you for bringing this to our attention. Upon further examination, we discovered that in Fig 4 (now renamed to Fig 5) the bar for “sonication alone” was indeed drawn inaccurately due to inputting the incorrect value in the graph-making software. Upon further inspection, we also found that the stated value of 27 *± 24 pg/rxn* was incorrect, and the correct value should have been 24 ± 17 pg/rxn. These corrections have been made in the manuscript (line 322) and in the figure itself. This revised value now accurately represents the size of the bar for “sonication alone”. We apologize for any confusion caused by this discrepancy. Please note that this figure has now been renumbered to Fig 5.

Furthermore, we have included the DNA yield value for the 100 °C boil condition (line 322).

**Reviewer comment 8**: *MTB cell concentration estimation for the clinical samples is not mentioned in the document, such detail could be useful to know the extent to which the optimized method used in this study for oral swabs spiked with 103 or 104 MTB cells holds good when clinical samples were considered.*

**Author response**: It was not possible to predict MTB cell concentrations in swabs prior to analysis. However, the values of 10^3^ to 10^4^ are consistent with measurements recently reported by Amy Steadman *et al* [2], Figure 1. While exact cell counts cannot be quantified, we would like to highlight that DNA quantities for each clinical swab are reported in the supplemental materials (S1 File).

**Reviewer comment 9**: *In ‘Results’ section, under the heading ‘Comparison of sensitivity between hybridization capture and QIA-EtOH in clinical oral swabs,’ the specificity values of hybridization capture and QIA-EtOH is not included, which could be helpful in comparing the false positive results.*

**Author response**: In this particular investigation, QIA-EtOH was not used to process any negative clinical samples. However, our consortium had previously conducted numerous studies (referenced at line 422) specifically aimed at characterizing the specificity of QIA-EtOH. Our focus in this paper was on characterizing the specificity of our hybridization capture method, which we observed to be an improvement over past specificity values obtained with QIA-EtOH.

**Reviewer comment 10**: *Also, why you did not consider all the 94 sputum Xpert Ultra-positive study participants altogether instead of considering only 30 of them, for the comparison of sensitivity between hybridization capture and QIA-EtOH in clinical oral swabs, since, on increasing the number of participants, the sensitivity for the hybridization capture was decreasing, then how to confirm that similar trend is also observed in the QIA-EtOH method in clinical oral swabs.*

**Author response**: Because clinical samples are a limited resource, it can be problematic to expend them on experiments involving previous methods. Therefore, the sensitivity comparison between hybridization capture and QIA-EtOH was limited to a subset of 30 samples. The primary goal of this subset comparison was to validate the compatibility of hybridization capture with clinical samples. Once this compatibility was confirmed, we extended the sensitivity analysis of hybridization capture to include the remaining 64 sputum Xpert Ultra-positive samples. Our consortium had already undertaken extensive studies (described in lines 71–75) characterizing the sensitivity of QIA-EtOH. Thus, we deliberately chose to allocate our time and resources to comprehensively characterize the sensitivity of hybridization capture.

**Reviewer comment 11**: *For the preparation of spiked oral swabs, you used lysis buffer containing 1% Triton-X-100 detergent. As Triton-X-100 itself is a lysing agent, it would be useful if you also include some experimental data representing the comparison of hybridization capture to QIA-EtOH in MTB-spiked oral swabs with and without 1% Triton-X-100 for the efficiency of the process on the DNA yield.*

**Author response**: We appreciate this suggestion. We have previously performed experiments comparing the DNA yield of hybridization capture in MTB-spiked oral swabs with and without 1% Triton-X-100. In these experiments we did not observe any difference in DNA yield between these two conditions. We speculate that the robustness of sonication may have obviated the need for the additional lysing power of Triton-X-100 for effective MTB lysis. To convey this information to the reader, we have included the corresponding data as a supplementary figure (S4 Fig).

**Reviewer comment 12**: *Under ‘Fig 4.’ description it was given that sonication with hybridization capture resulted in a ~100-fold increase in DNA yield compared to QIA-EtOH extraction alone. Similarly, under ‘discussion’ section it was given that sonication prior to Qiagen DNA extraction yielded a 100-fold increase in DNA yield compared to QIA-EtOH extraction alone. If sonication showing 100-fold increase in DNA yield with both Hybridization capture and with QIA-EtOH, then how your developed method is better than Qiagen apart from cross contamination protection, since, it would be much easier to use sonication with QIA-EtOH then sonication with hybridization capture which requires designing of probes for specific target sequences.*

**Author response**: We appreciate this observation. In addition to being less susceptible to cross-contamination, hybridization capture is in fact easier than QIA-EtOH when targeting a specific known pathogen.

As referenced in lines 434–436, hybridization capture is an established technique that is used in automated platforms for nucleic acid capture/amplification, including Nuclein’s DASH platform. Automation is not possible with EtOH precipitation. Therefore, we consider our hybridization capture protocol for oral swabs as a proof-of-principle and a stepping-stone towards automated approaches using this methodology.

With regard to the need to design probes, it needs to be done only once for each pathogen. Once it is reported here, it does not need to be done again for *M.* *tuberculosis*.

**Reviewer comment 13**: *It would be useful if you include some cost comparison between the above two methods.*

**Author response**: Thank you for this valuable suggestion. We have included a cost comparison between the two methods in the discussion (lines 426–428), which states: “Notably, this improved performance was achieved without a significance difference in total reagent cost ($4.50 for hybridization capture vs. $4.00 for QIA-EtOH at the time of publishing).”

**Reviewer # 2:**

**Reviewer comment 14**: *The authors have mentioned several methods and modifications (proteinase K, sonication, hybridization capture etc) which have improved the DNA isolation from oral swabs. However (in all experiments Fig 2, 3 and 4), no statistics has been applied to ascertain whether this improvement was significant or not? How many samples were included in these experiments? Three? This is especially important as the same difference was not seen when applied to real samples and not spiked samples.*

**Author response**: Thank you for bringing up this important point. The number of samples included in these experiments is specified in the figure caption. Three samples were included in the experiments for Fig 2 (renamed to Fig 3) and Fig 3 (renamed to Fig 4), while eight samples were included in experiments for Fig 4 (renamed to Fig 5). These sample numbers can be found in lines 290, 311, and 332 respectively. In response to your suggestion, we have now added brackets with P-values to these figures to visually indicate the statistical significance of each modification. Please note, in this latest submission these figures have been renumbered from “2, 3, and 4” to “3, 4, and 5” respectively.

**Reviewer comment 15**: *Line 246-247. “Samples were identified as positive if any amplification (above the threshold) was detected after 45 cycles”. Please clarify this. For qPCR any amplification (above the threshold) was detected after 45 cycles?*

**Author response**: Thank you for bringing attention to this point. It appears there was a typographical error. The correct statement should read: “Samples were identified as positive if any amplification (above the threshold) as detected **before** 45 cycles.” This change is reflected in the manuscript in line 262.

**Reviewer comment 16**: *Pg 9. Please include a workflow for the whole study in accordance with the STARD guidelines defining the Cohort 1 and cohort 2. The protocols can also be included in work flow format.*

**Author response**: Good suggestion, we have added a STARD diagram (Fig 2).

**Reviewer comment 17**: *How was sensitivity and specificity calculated? Xpert Ultra as gold standard? I am unable to align the results obtained with the supplementary data provided. Were the results of both the Cohorts combined for analysis? Was culture data also compared for analysis?*

**Author response**: We apologize for the lack of clarity here. Reference standard tests are reported in the methods section ‘Collection of clinical samples and test assignment’ but we have clarified reporting of the cohort breakdowns and respective standards used, with both the inclusion of a STARD diagram (Fig 2) and modifications to the text.

The following text (226–230) should clarify that Xpert Ultra was used as the standard for sensitivity and Xpert Ultra and culture were used as the standard for specificity: “In the second round of analysis, the remaining 64 participants determined to be positive by sputum Xpert Ultra in Cohort 1 and 42 participants negative by sputum Xpert Ultra and culture in Cohort 2 were assigned only one random swab for processing by hybridization capture for evaluation of sensitivity and specificity respectively (Fig 2).”

**Reviewer comment 18**: *Please mention that study was done as a demonstration study, wherein results of the samples were known prior to performing the assay.*

**Author response**: Thank you for this suggestion. The results of the samples were not known prior to performing the assay. We have instead added the following statement (lines 232­–233), which better describes the study strategy: “The study was conducted as a demonstration study, wherein the TB statuses of participants who provided the swab samples were known prior to performing the assays.”

**Reviewer comment 19**: *The authors should shorten the discussion and mention strengths and limitations of the study.*

**Author response**: The discussion has been shortened.

We have added limitations of the study including lines 407–410 and lines 417–419 which state the following respectively: “Limitations of this study may also contribute to a greater proportion of fragile MTB cells and/or cell-free DNA in clinical samples. These include the collection of samples post-TB treatment (up to 72 hours) and the processing of samples following a freeze-thaw cycle, which is known to disrupt bacterial integrity” and “This study is also limited in applicability to other settings and populations. Evaluation of other cohorts is warranted.”

Additionally, lines 410–412 now highlights an additional strength of our study, namely that the clinical samples used to compare the sensitivity of QIA-EtOH and hybridization capture were paired swabs from the same donor. The added lines read as follows: “While QIA-EtOH was tested on a small sample size of 30, our study is strengthened by testing two paired swabs per participant, one for each method.”

**Reviewer # 3:**

**Reviewer comment 20**: *The authors didn't provide the clinical details of the samples included in the study for evaluation of the samples.*

**Author response**: Clinical details are provided by way of cohort definitions, which note the microbiological reference standard used for each cohort (lines 217–221). Please see our new STARD diagram (Fig 2) for further clarification.

**Reviewer comment 21**: *To evaluate the specificity the authors needs to also include the sample from similar diseased control of non-TB patients to check the cross reactivity.*

**Author response**: For Cohort 2 of this study, the enrollment criteria included participants with symptoms consistent with TB (lines 218-221). Among these samples, swabs from participants who were later found to be TB-negative via Xpert Ultra and culture were tested by hybridization capture to evaluate specificity.

**Reviewer comment 22**: *The formatting of manuscript is poorly done and in between the paragraph/test figure and table legends inserted.*

**Author response**: We apologize for any difficulties reading the manuscript due to formatting. However, the formatting of the manuscript was done in-line with the submission guidelines outlined by PLOS ONE. This includes having the figure legends inserted between paragraphs, with the figures themselves in a separate file. If we have misinterpreted the meaning, we welcome the opportunity to respond again.

**Reviewer comment 23**: *Need revision of minor spelling corrections in the manuscript.*

**Author response**: Thank you for pointing this out. We have addressed minor spelling corrections throughout the manuscript.

**Reviewer comment 24**: *Authors should include the table for clinical details of samples tested and mentioning the sensitivity and specificity of tested samples for better representation of data.*

**Author response**: We previously included the results of the microbiological reference standard in the supplemental figure (S1 File) with our test results. Rather than move that figure to the text we think it’s better suited to remain in the supplemental figures. We also clarified how the sensitivity and specificity were calculated with modifications to the text where we define the clinical sample collection and test allocation. See also our response to other reviewer suggestions above.

**Works Cited:**

1.	Luabeya AK, Wood RC, Shenje J, Filander E, Ontong C, Mabwe S, et al. Noninvasive Detection of Tuberculosis by Oral Swab Analysis. J Clin Microbiol. 2019 Feb 27;57(3):e01847-18.

2.	Steadman A, Andama A, Ball A, Mukwatamundu J, Khimani K, Mochizuki T, et al. New manual qPCR assay validated on tongue swabs collected and processed in Uganda shows sensitivity that rivals sputum-based molecular TB diagnostics [Internet]. medRxiv [Preprint]; 2023 [cited 2024 Jan 25]. Available from: https://www.medrxiv.org/content/10.1101/2023.08.10.23293680v1
