# Supplementary material for: Detection of Mycobacterium tuberculosis from tongue swabs using sonication and sequence-specific hybridization capture
Source: PLoS One. 2024 Aug 15;19(8):e0308235. doi: 10.1371/journal.pone.0308235 (PMC11326604; doi:10.1371/journal.pone.0308235)
Supplement: Attachment — Submitted filename: Response_To_Reviewers_2024_07_08.docx [file pone.0308235.s008.docx]

**Response to Reviewers**

Dear PLOS ONE reviewers,

Thank you for the opportunity to resubmit our manuscript titled “Detection of *Mycobacterium tuberculosis* from tongue swabs using sonication and sequence-specific hybridization capture” for publication in PLOS ONE. We appreciate the time and effort dedicated to providing feedback on our manuscript, and we have made the following changes to our submission to address the feedback provided. These changes are found in the revised manuscript file with tracked changes. Please see below, in blue, for a point-by-point response to the reviewers’ comments and concerns. All line numbers refer to the clean revised manuscript file **without** tracked changes.

**Reviewers' Comments to the Authors:**

**Reviewer # 3:**

**Reviewer comment 1**: *“As the previously advised to include the clinical details of subjects enrolled in the study but in revised manuscript the data is missing.”*

**Author response:** We appreciate the reviewer’s reminder to include the baseline characteristics of the participants in our study. Since the initial submission of our manuscript, our group has published a related manuscript by Wood et al. [1] which evaluated swabs from the same group of participants. This published manuscript includes a table (Table 1) that describes the baseline characteristics of the participants from cohorts 1 and 2 including age, gender, race, HIV, status, and other relevant clinical details.

In light of this, we have added a sentence to the revised manuscript (lines 221­–223) indicating that the clinical details of the subjects enrolled in this study can be found in the manuscript by Wood et al. [1]. We believe this provides the necessary information and maintains consistency between our publications.

**Reviewer comment 2:** *“Moreover the cohort 1 and 2 are not clearly defined. Inclusion and exclusion criteria not mentioned for subject recruitment.”*

**Author response:** We appreciate the reviewer’s concern regarding the definition of cohorts 1 and 2. While we have previously defined these cohorts in our manuscript (lines 217–221), we understand the need for more detailed descriptions.

The Wood et al. [1] study provides comprehensive definitions of cohorts 1 and 2, including detailed inclusion and exclusion criteria. We added a reference to this publication in our manuscript for those who require further information. Specifically, we have added the following sentence to the methods section (lines 221–223):

*"Detailed definitions of Cohorts 1 and 2, including participant baseline characteristics and inclusion and exclusion criteria, are provided in Wood et al."*

**Reviewer comment 3: “***Comparison of test with culture finding not calculated.”*

**Author response:** Thank you for your comment regarding the use of sputum GeneXpert Ultra to determine TB-positive samples in our study.

While sputum culture is traditionally regarded as the gold standard for TB diagnosis, the GeneXpert Ultra assay is endorsed by the World Health Organization (WHO) [2] and is considered a valid method for bacteriologically confirming TB cases. As stated by the WHO: “People diagnosed with TB using **rapid molecular tests** recommended by the WHO, lateral flow urine lipoarabinomannan (LF-LAM) assays, sputum smear microscopy or culture are defined as 'bacteriologically confirmed' cases of TB” [3] .

However, we acknowledge the importance of including culture data for comprehensive validation. To address this concern, we will revise our manuscript to include a statement acknowledging this limitation. Specifically, we have added the following sentence to the discussion of limitations in lines 413­–414:

*“Our study is also limited in that culture data was not collected for Xpert Ultra-positive participants.”*

We hope this clarification and the addition of the limitation statement to our manuscript address your concerns.

**Reviewer comment 4: “***Health control other than patients not included in the study as this is crucial part to evaluate/validate any test for disease detection.”*

**Author response:** We understand the concern regarding the selection of control subjects in our study. However, we would like to highlight a couple points that justify our methodology.

Our choice to include symptomatic but TB-negative individuals in our control group was made deliberately to avoid inaccurately reporting our specificity due to spectrum bias. Spectrum bias is the misrepresentation of a test’s performance due to a study including too narrow a range of individuals with or without the disease of interest [4]. A review of meta-analyses of diagnostic tests [5] found that including severe cases and healthy controls resulted in a relative diagnostic odds ratio (RDOR) of 4.9, indicating a considerable overestimation of diagnostic accuracy due to the extreme contrast between groups. Using fully healthy controls (i.e., no TB-like symptoms) would likely lead to fewer false positive results, thus overestimating the specificity.

Our inclusion of symptomatic, TB-negative subjects as controls aligns with the recommendations from the STARD guidelines [6] and PRISMA which state that primary studies of diagnostic test accuracy should recruit individuals that represent the population in which the test will be typically used. The primary purpose of our test is to distinguish between TB-positive and TB-negative individuals in a clinical setting where TB is suspected, which includes patients presenting with TB-like symptoms. Including truly healthy controls (individuals without any symptoms) would not accurately reflect the clinical scenarios in which the diagnostic test is applied.

In conclusion, we believe that the inclusion of symptomatic TB-negative controls is a methodologically sound approach that enhances the relevance and applicability of our findings. We hope this clarification addresses your concerns.

**Works Cited:**

1.	Wood RC, Luabeya AK, Dragovich RB, Olson AM, Lochner KA, Weigel KM, et al. Diagnostic accuracy of tongue swab testing on two automated tuberculosis diagnostic platforms, Cepheid Xpert MTB/RIF Ultra and Molbio Truenat MTB Ultima. Journal of Clinical Microbiology. 2024 Mar 14;62(4):e00019-24.

2.	Organization WH. WHO consolidated guidelines on tuberculosis: module 3: diagnosis: rapid diagnostics for tuberculosis detection [Internet]. World Health Organization; 2021 [cited 2024 Jul 1]. Available from: https://iris.who.int/handle/10665/342331

3.	2.2 Diagnostic testing for TB [Internet]. [cited 2024 Jul 1]. Available from: https://www.who.int/teams/global-tuberculosis-programme/tb-reports/global-tuberculosis-report-2023/tb-diagnosis---treatment/2.2-diagnostic-testing-for-tb

4.	Usher-Smith JA, Sharp, SJ, Griffin SJ. The spectrum effect in tests for risk prediction, screening, and diagnosis. BMJ. 2016 Jun 22;353:i3139.

5.	Rutjes AWS, Reitsma JB, Di Nisio M, Smidt N, van Rijn JC, Bossuyt PMM. Evidence of bias and variation in diagnostic accuracy studies. CMAJ. 2006 Feb 14;174(4):469–76.

6.	Bossuyt PM, Reitsma JB, Bruns DE, Gatsonis CA, Glasziou PP, Irwig L, et al. STARD 2015: an updated list of essential items for reporting diagnostic accuracy studies. BMJ. 2015 Oct 28;351:h5527.
